# Use of Proviral DNA to Investigate Virus Resistance Mutations in HIV-infected Zimbabweans

**DOI:** 10.2174/1874285801711010045

**Published:** 2017-04-28

**Authors:** Tutsirai V. Musingwini, Danai T. Zhou, Doreen Mhandire, Kerina Duri, Exnevia Gomo, Olav Oktedalen, Benjamin Chimukangara, Tinei Shamu, Sandra Shawarira-Bote, Collet Dandara, Babill Stray-Pedersen

**Affiliations:** 1 University of Zimbabwe, College of Health Sciences, Department of Medical Laboratory Sciences, Harare, Zimbabwe; 2 Institute of Clinical Medicine, University in Oslo, Oslo University Hospital, Oslo, Norway; 3 University of Zimbabwe, College of Health Sciences, Department of Chemical Pathology, Harare, Zimbabwe; 4 Universisty of Zimbabwe, College of Health Sciences, Department of Immunology, Harare, Zimbabwe; 5 Department of Infectious Diseases, Oslo University Hospital, Oslo, Norway; 6 Africa Centre for Health and Population Studies, University of KwaZulu-Natal, KwaZulu-Natal, South Africa; 7 Newlands Clinic, Newlands, Harare, Zimbabwe; 8 Division of Human Genetics, Department of Clinical Laboratory Sciences & Institute for Infectious Disease and Molecular Medicine, Faculty of Health Sciences, University of Cape Town, Cape Town, South Africa; 9 Institute of Clinical Medicine, University in Oslo and Women’s Clinic, Oslo University Hospital, Oslo, Norway

**Keywords:** Drug resistance, Genotyping, HIV proviral DNA, Sequencing, Zimbabwe

## Abstract

**Background:**

Antiretroviral therapy (ART) to suppress HIV replication has reduced morbidity and mortality yet effectiveness of current HIV drugs is threatened by HIV drug resistance (HIVDR) mutations.

**Objective:**

To determine HIVDR mutations using proviral DNA from specimens of patients presenting to an HIV treatment clinic.

**Methods:**

DNA from 103 patients, 86 treatment-experienced, 17 treatment-naïve, were genotyped for the HIV-1C reverse transcriptase gene (RT; codons 21-304) using Sanger sequencing and sequences analyzed using Sequencher software. Resistance mutations were interpreted using Stanford HIVDR reference database.

**Results:**

Median age was 39 (IQR, 33-46) years and 80% of patients were female. Six-percent (n=6) had at least one HIVDR mutation, comprising NRTI-associated mutations, (M184V, T69D, T69N and V75I); NNRTI-associated mutations (G190A, K103N, V106M, Y181C) and thymidine analogue associated mutations (D67N, K70R, K219Q, L210W, M41L, T215Y). Of the six participants, with at least one HIVDR mutation, all were treatment experienced, five were on tenofovir, lamivudine and nevirapine and one was on tenofovir, lamivudine and atazanavir. There was no difference in median CD4 count and viral loads when patients were compared by presence of HIVDR mutations.

**Conclusion:**

We demonstrated the use of proviral DNA in HIVDR testing in adult patients and present that all the patients with various kinds of HIVDR mutations were treatment experienced, pointing to the role of drug regimens in driving viral mutations. Thus, the use of proviral DNA has potential to help provide surveillance on risk of HIVDR in HIV-infected individuals who are on treatment, which may assist in corrective treatment.

## INTRODUCTION

Historically, the human immunodeficiency virus (HIV) has infected nearly 78 million people and close to 39 million have died of acquired immunodeficiency syndrome (AIDS), to date [1, 2]. According to 2013 estimates, the adult prevalence of HIV in Zimbabwe was 15% affecting 1.2 million adults and 170 000 children (0-14 years) [3]. In an effort to curb the pandemic, the World Health Organisation (WHO) has recommended the use of combination antiretroviral therapy (cART), with first-line regimens in adults having two nucleoside reverse-transcriptase inhibitors (NRTIs) and a non-nucleoside reverse-transcriptase inhibitor (NNRTI). Ritonavir-boosted protease inhibitors (bPIs) are preferred in second-line regimens, in place of NNRTIs [4, 5].

 In line with these recommendations, the Zimbabwean government through the Ministry of Health and Child Care (MOHCC) first introduced the national antiretroviral treatment (ART) program in 2004, which was providing approximately 581 801 adults with ART by 2012 [5, 6]. Estimates showed that 962 779 people including 104 937 children were in need of ART in Zimbabwe in 2013 (based on CD4 counts ≤ 350 cells/ml), and projections showed that the number of people in need of ART will increase to 1.3 million and 1.4 million in 2014 and 2015, respectively [5]. With the rapid scale up of ART over the years, the emergence of antiretroviral (ARV) drug resistance mutations in Zimbabwe has been inevitable as in any other settings [7, 8]. Treatment failure may result from acquired drug resistant mutations due to ARV drug pressure and such variants of the virus can also be transmitted to newly infected individuals who are not yet on ART as transmitted drug resistance [9, 10].

Findings from the PharmAccess African Studies to Evaluate Resistance Monitoring (PASERM) among 2 436 antiretroviral-naive individuals in 11 geographic areas in Kenya, Nigeria, South Africa, Uganda, Zambia and Zimbabwe showed overall baseline prevalence of acquired drug resistance mutations (DRMs) of 5.6% [CI: 4.6 - 6.7] in 2007-2009 [11]. The most commonly occurring mutations were the NNRTI mutation K103N (1.8%), thymidine analog mutation (TAM): M41L (1.6%) and NRTI mutation M184V (1.2%).

 TAMs result in multi-nucleoside resistance [12, 13], K103N and Y181C mutations cause high-level resistance to efavirenz (EFV) and nevirapine (NVP) [14], whilst M184V causes high-level resistance to lamivudine (3TC) and rapidly emerges in patients on 3TC regimens [12-15]. Thus, the presence of such viral mutations reduces viral susceptibility to ARV drugs, in comparison with wild type virus and will eventually compromise effectiveness of ART [16].

In a recent study on acquired ARV drug resistance mutations in treatment experienced patients in Zimbabwe, 11 of 108 (10%) patients had primary drug resistance mutations, with the mutation M184V occurring most frequently [17]. Another study on children aged 0 to 18 months in a prevention of mother to child transmission (PMTCT) program in Zimbabwe, showed that 29 (12.5%) children had NRTI-associated mutations and 145 (62.5%) had NNRTI-associated mutations [18]. Transmission of such viral variants to newly infected individuals could jeopardize the effective use of the already limited ART regimens in Zimbabwe.

It is necessary to determine the HIV drug resistance mutations in a population if management of HIV/AIDS through ARV therapy is to remain effective. Although sequencing of plasma viral RNA (vRNA) remains the gold standard in genotypic resistance testing [19], the method has been shown to miss the presence of drug resistance if there has been poor suppression of the virus due to interruption in adherence. Moreover, some studies have shown analysis of the alternative proviral DNA to give relatively similar results to plasma vRNA [19, 20] which warrants the investigative use of proviral DNA in drug resistance testing. Hence the main objective of this study was to determine HIVDR mutations using proviral DNA from blood specimens of HIV infected ART-naïve and ART-experienced patients presenting to an HIV treatment clinic in Harare, Zimbabwe.

## MATERIALS AND METHODS

### Participant recruitment

One hundred and three (103) HIV-infected adult patients visiting the HIV clinic in Harare, Zimbabwe for treatment during the period March to August 2013 were recruited for the study. Details of the study site have been described previously [21]. Some of the participants were ART-experienced and the control group was ART-naive. ART-naïve group acted as a control group, to discount effect of proviral DNA archived in patients’ cells. Patients were invited to join the study as they came and written informed consent was obtained from all participants. A questionnaire-guided interview was conducted to obtain demographic and clinical data such as age, sex, drug regimen, duration on ART and time since diagnosis. The study was ethically cleared by the Joint Research Ethics Committee of the University of Zimbabwe College of Health Sciences and the Parirenyatwa Group of Hospitals (JREC), the Medical Research Council of Zimbabwe (MRCZ) and Research Ethics Committee, Norway (REK) [21].

### Specimen Collection

Venipuncture was used to draw 5 ml of whole blood from each participant into EDTA-coated tubes labeled appropriately. Routine CD4 cell counts were done using Partec Cyflow Counter II and viral loads were measured using absolute quantification by digital PCR methods on a Roche LightCycler^®^ 480 System at clinic site. Samples were couriered to African Institute of Biomedical Science and Technology (AiBST) in Harare, Zimbabwe and stored at -20°C prior to viral genotyping.

### DNA Extraction

DNA extraction was achieved using the QIAGEN QIA amp DNA mini extraction kit (QIAGEN, GmbH Germany) according to manufacturer’s protocol. In summary, 20 µl of QIAGEN protease was mixed with 200 µl of thawed whole blood. 200 µl of buffer was added to the sample tubes and vortexed for 15 seconds. Samples were then incubated at 56^o^C for 10 minutes. 200 µl of ethanol was added to the lysate and mixed by vortexing for 15 seconds. The lysate was transferred to silica membrane spin columns and was washed using provided wash buffers. DNA was recovered using an elution buffer and the extract was stored at -20°C before use in the polymerase chain reaction (PCR).

### Polymerase Chain Reaction

PCR conditions, primers and reagent volumes were adopted from a previously published study [17] and details of primers are shown in (Tables **1** and **2**). In summary, DNA was amplified by nested PCR using Thermo Scientific Phusion Hot Start II High-Fidelity DNA polymerase (Affibody AB, Sweden) on a PTC-100 thermo cycler (BioRad, California, USA).

Cycling conditions were as follows: 96°C for 2 minutes; 40 cycles of 96°C for 20 seconds; 55°C for 20 seconds; and 72°C for 2 minutes, with a final extension at 72°C for 10 minutes. Second round PCR conditions were the same as the first round, with 35 cycles and an annealing temperature of 56^o^C. Amplification was confirmed by gel electrophoresis before purification.

### Polymerase Chain Reaction Product Purification and Sequencing

The QIAquick PCR purification kit (QIAGEN, GmbH, Germany) was used to purify the amplicons in preparation for sequencing, according to manufacturer’s instructions. Briefly, 20μl of second round amplicon was mixed with 100μl of binding buffer (1:5 ratios). The mixture was transferred to a spin column and centrifuged at 13000rpm for 1 minute. The columns were washed with 20μl washing buffer. Purified amplicon was eluted in 50μl of elution buffer. Amplicons were sequenced at Molecular Cloning Laboratory (MCLAB, California, USA) on an ABI 3730xl genetic analyzer. The sequencing primers used are shown in (Table **1**). Sequence assembly was done using Sequencher v.5.3 (Gene Codes Co.) and drug resistance mutations were determined using the Stanford HIV drug resistance database (Stanford HIVdb) [22].

## RESULTS

### Demographic Characteristics

A total of 103 patient samples were analyzed for drug resistance mutations in this study. Of these patients, 86 (83%) were receiving ART, and 17 were ART-naïve. The median age was 39 (33-46) years, and the median time on ART was 4 (2-7) years. Time since diagnosis was longer for patients receiving ART than those who were ART-naïve (p=0.02) (Table **3**).

### HIV Drug Resistance Mutations

Six of the 103 participants (6%) had drug resistance mutations (Table **4**). The mutations observed were; NRTI mutations T69D, T69N, V75I and M184V; NNRTI mutations, K103N, V106M, Y181C and G190A; and TAMs (M41L, D67N, K70R, L210W, T215Y, K219Q) (Table **3**).

One (0.97%) of the samples analyzed had only one NRTI mutation (T69N); two of the samples had three drug resistance mutations each, and shared a common NRTI mutation M184V. One participant had seven mutations, three were TAMs (L210W, T215Y and M41L), two were NRTI mutations (M184V, V75I) and two were NNRTI mutations (G190A, V106M) (Table **4**). The participant with seven mutations had been on ART for 5 years, had a CD4 cell count of 399 cells/µl, as determined soon after recruitment, but the patient died during the course of the study. The most frequently occurring mutation in the whole group of patients was the M184V mutation, which occurred in five of the six (83%) participants who had mutations (Table **4**).

## DISCUSSION

Drug resistance testing, before initiating ART, is not currently performed, as a routine test, in resource-limited settings, mainly due to costs. The current universal standard specimen in drug resistance testing is plasma. Plasma samples must be stored at a temperature of -80°C within 6 hours of collection. The costs incurred in transportation, storing, extraction and reverse transcribing vRNA has made resistance testing from plasma inaccessible in settings where resources are limiting [19]. Whilst genotypic testing is reliable in patients with high viral loads (>1000 copies/ml), access to viral load testing is also not widely available in Zimbabwe due to the costs associated with the assay [5, 17].

A cost effective method of genotypic drug resistance testing using proviral DNA was used in this study, to inform about HIVDR mutation patterns in HIV patients. A similar study carried out in Zimbabwe demonstrated that HIV genotypic testing from whole blood is more affordable in resource limited settings (~$34 per sample excluding indirect costs) in comparison with currently existing methods, with unit cost ranging from $250 -500 [23, 24].

Of the 103 samples that were successfully sequenced, six had drug resistance mutations. The most frequently occurring mutation was M184V, which was observed in five out of the six sequences (Table **4**). This is consistent with various studies done in Zimbabwe [17, 19, 20]. M184V causes high level resistance to 3TC [15, 19], a drug that is recommended in all 1^st^ and 2^nd^ line ART regimens in Zimbabwe [5]. However, the M184V mutation causes increased susceptibility to stavudine (d4T) and TDF [15, 19], increases the *in vitro* susceptibility to zidovudine (AZT) and d4T, delays the appearance of TAMs and reduces viral replicative capacity [19-26]. Hence any regimen consisting of TDF, AZT or d4T plus 3TC has been shown to be effective in the presence of the M184V mutation, making 3TC a continued drug of choice despite presence of the M184V mutation.

Four of the viral sequences (67%) had at least one TAM. TAMs are drug resistance mutations which reduce antiviral activity of thymidine analogues d4T and AZT [26]. In this study, TAMs observed were M41L, D67N, K70R, L210W, T215Y and K219Q. Studies have shown TAMs to appear through two pathways in a particular order: TAM-1 (M41L/L210W/T215Y) and TAM-2 (D67N/K70R/T215F/K219Q) [26]. Reverse transcriptase (RT) of HIV-1 Subtype C has been seen to accumulate TAM-2 pathway mutations (e.g. T215F) first, whilst the RT of HIV-1 Subtype B follows TAM-1 pathway (e.g. T215Y) mutations [27, 28]. However, it remains that the two pathways are not mutually exclusive [29]. One of the sequences had all TAM-1 mutations, and another had all TAM-2 mutations Table **4** Two of these TAMS: D67N and T215K were also observed in a study recently carried out in Zimbabwe [17]. TAMS mainly occur less commonly in patients receiving regimens with TDF and abacavir (ABC) [30]. Initiating treatment with ABC and TDF as part of the drug regimen may reduce occurrence of these mutations.

The WHO resistance classification considers an HIVDR prevalence of 5-15% to be moderately high in treatment naïve patients [31]. Of the 103 patient samples analyzed in this study, 17 were ART-naïve and there were no drug resistance mutations observed in any of these samples. Over 5% in treatment naïve patients is reflective of high levels of transmitted resistance which is common in the US and Europe. There are generally 0 to <5% rates of transmitted resistance in sub-Saharan Africa as reported by Soo Yon Rhee *et al*., 2015 [32].

Among the six patient samples with drug resistance mutations, the CD4 counts ranged from 106 cells/ml to 988 cells/ml, while viral loads ranged from undetectable to 131 copies/ml for four of the six patients who had viral load results. High viral loads were not associated with accumulation of drug resistance mutations (P >0.05) in this study. Moreover, lower CD4 counts and demographic characteristics were not significantly associated with development of resistance mutations (Table **4**).

In Zimbabwe, the first line regimen comprises of TDF, 3TC, and EFV. D4T is being phased out with its use being currently limited to cases intolerant of either zidovudine or TDF. Protease inhibitors are favored in the second line regimen and third line regimens for infants [5]. Despite the presence of drug resistance mutations in six of the patient samples, the current recommended drug regimens are still effective in suppressing viral replication, as the mutations did not confer significant levels of resistance to the current recommended regimens. Genotypic testing using proviral DNA is a potential low-cost method that can be used in surveillance monitoring of drug resistance in resource limited settings like Zimbabwe [33]. Genotypic testing may also be made cheaper by use of dried blood spots, which are easier to collect, transport, store and process.

## CONCLUSION

As drug resistance mutations were detected in 6% of patients, despite the viral load threshold of >200 copies/ml being met, there is, need to monitor treatment experienced patients periodically for HIVDR mutations. The sustainability of the achievements of ART not only relies on close clinical monitoring of disease progression, but in resource limited settings, there is need to avail affordable treatment monitoring methods. Such monitoring methods will help assess the extent and if any, risks to the population of drug resistance, which allows decision making on drug regimens which best, suppress viral replication.

## Figures and Tables

**Table 1:** Details of primers.

First round PCR primers were as follows:
Pro1: 5’-CAGAGCCAACAGCCCCACCA-3’ (forward) and
BC21: 5’-CTGTATTTCAGCTATCAAGTCTTTTGATGGG-3’ (reverse).
Second round PCR primers included:
M13_Pol1: 5’-GTTAAACAATGGCCATTGACAG-3’ (forward) and
BC20: 5’-CTGCCAATTCTAATTCTGCTTC-3’ (reverse), for amplifying an 849 bp reverse transcriptase gene (RT), spanning codons 21 - 304.

**Table 2:** HIV-1 reverse transcriptase sequencing primers.

Name	Sequence	Direction	HXB2 Position
RTC1F	5’-ACCTACACCTGTCAACATAATTG-3’	Forward	2486-2508
RTC3F	5’-CACCAGGGATTAGATATCAATATAATGTGC-3’	Forward	2956-2994
RTC4R	5’-CTAAATCAGATCCTACATACAAGTCATCC-3’	Reverse	3129-3101

**Table 3:** Demographics and clinical characteristics of study participants.

**Characteristics of the 103 Patients**				
**Variable**	**All (N=103)**	**Treatment experienced (n=86)**	**Treatment naïve (n=17)**	**p-value**
**Demographics**				
Females/number (%)	82 (80)	71 (87)	11 (13)	0.095
Males /number (%)	21 (20)	15 (71)	6 (29)	
Age in years /median (IQR)	39 (33-46)	39.5 (35-46)	36 (31-40)	0.197
**Clinical Characteristics**				
Time on ART in years /median(IQR)		4(2-7)		
Time since HIV diagnosis in years /median (IQR)	5 (3-8)	5 (3-8)	2 (1-4)	<0.0001
CD4 count/ median(IQR)	468 (269- 658)	491 (294- 662)	292 (4-540)	0.056
**Patient Characteristics by Drug Resistance Mutations**				
**Variable**	**All (N=103)**	**HIVDR^+^** **(n=6), 6%**	**HIVDR^-^ (n=97), 94%**	**p-value**
Age in years /median (IQR)	39 (33-46)	45 (32-55)	39 (33.5-45)	0.852
CD4 count cells /mm^3^/ median (IQR)	468 (269-658)	261 (127-399)	480 (281-661)	0.117
Viral load/ copies/ml / median /(IQR)	37 (20-37)	37 (20-45)	37 (20-37)	0.688
Time on ART in years/ median(IQR)	3 (0.1-5)	4 (1.5-5)	3 (0.05-5. 5)	0.232

ART, antiretroviral therapy; NNRTI, non-nucleotide reverse transcriptase inhibitor; PI, protease inhibitor; IQR, Interquartile range; HIV, Human immunodeficiency virus; CD4,Cluster of differentiation 4, p values* obtained using Pearson chi squared tests, p values** obtained using median tests, HIVDR^+^, HIV drug resistance mutations; HIVDR^-^ No HIV drug resistance mutations.

**Table 4:** Observed HIV-1 subtype C drug resistance mutations.

**Patient ID**	**Drug Regimen**	**Viral Load** **(copies/ml)**	**CD4 count** **(cells/ml)**	**Years on ART**	**Mutations**			
					**NRTI**	**NNRTI**	**TAMs**	**Total**
48	**1**	LFU	172	4	M184V	K103N	K219Q	3
54	**2**	20	106	1.5	M184V T69D		D67N	3
91	**1**	131	127	5	M184V	Y181C		2
172	**1**	20	988	14	M184V		D67N K219Q K70R	4
185	**1**	20	351	5	T69N			1
196	**1**	LFU	399	5	M184V V75I	G190A V106M	L210W T215Y M41L	7

1=TDF/3TC/NVP 2= TDF/ATV/3TC, TDF, Tenofovir; 3TC, Lamivudine; NVP, Nevirapine; ATV, Atazanavir ; NRTI, nucleoside reverse transcriptase inhibitor; NNRTI, non-nucleoside reverse transcriptase inhibitor; TAMs, Thymidine analogue mutations;CD4,cluster of differentiation 4; LFU, Lost to follow-up.
